# Pt nanoparticles decorated heterostructured g-C_3_N_4_/Bi_2_MoO_6_ microplates with highly enhanced photocatalytic activities under visible light

**DOI:** 10.1038/s41598-019-42973-6

**Published:** 2019-05-21

**Authors:** Z. Jia, F. Lyu, L. C. Zhang, S. Zeng, S. X. Liang, Y. Y. Li, J. Lu

**Affiliations:** 1 Hong Kong Branch of National Precious Metals Material Engineering Research Center, Department of Material Science and Engineering, City University of Hong Kong, Hong Kong, China; 2 Department of Mechanical Engineering, City University of Hong Kong, Hong Kong, China; 3 School of Engineering, Edith Cowan University, 270 Joondalup Drive, Joondalup, Perth, WA 6027 Australia; 4 Center of Super-Diamond and Advanced Films (COSDAF), City University of Hong Kong, Kowloon, Hong Kong China; 5 Department of Physics and Materials Science, City University of Hong Kong, Kowloon, Hong Kong China; 6 Centre for Advanced Structural Materials, City University of Hong Kong, Shenzhen Research Institute, 8 Yuexing 1st Road, Shenzhen Hi-Tech Industrial Park, Nanshan District, Shenzhen, China

**Keywords:** Pollution remediation, Photocatalysis

## Abstract

Exploring an efficient and photostable heterostructured photocatalyst is a pivotal scientific topic for worldwide energy and environmental concerns. Herein, we reported that Pt decorated g-C_3_N_4_/Bi_2_MoO_6_ heterostructured composites with enhanced photocatalytic performance under visible light were simply synthesized by one-step hydrothermal method for methylene blue (MB) dye degradation. Results revealed that the synthetic Pt decorated g-C_3_N_4_/Bi_2_MoO_6_ composites with Bi_2_MoO_6_ contents of 20 wt.% (Pt@CN/20%BMO) presented the highest photocatalytic activity, exhibiting 7 and 18 times higher reactivity than the pure g-C_3_N_4_ and Bi_2_MoO_6_, respectively. Structural analyses showed that Bi_2_MoO_6_ microplates were anchored on the wrinkled flower-like g-C_3_N_4_ matrix with Pt decoration, leading to a large expansion of specific surface area from 10.79 m^2^/g for pure Bi_2_MoO_6_ to 46.09 m^2^/g for Pt@CN/20%BMO. In addition, the Pt@CN/20%BMO composites exhibited an improved absorption ability in the visible light region, presenting a promoted photocatalytic MB degradation. Quenching experiments were also conducted to provide solid evidences for the production of hydroxyl radicals (^•^OH), electrons (e^−^), holes (h^+^) and superoxide radicals (^•^O^2−^) during dye degradation. The findings in this critical work provide insights into the synthesis of heterostructured photocatalysts with the optimization of band gaps, light response and photocatalytic performance in wastewater remediation.

## Introduction

Advanced oxidation processes (AOPs) with their superior purification efficiency of toxic organic compounds have been recently attracted large attentions in industrial wastewater treatment^1–6^. Compared to alternatives, such as Fenon/Fenton-like process^7–9^ and ozone oxidation^10–12^, semiconductor photocatalysts have received tremendous attentions to address the worldwide environmental and energy concerns^13^. Since Fujishima and Honda discovered the photoelectrochemical effects of TiO_2_^14^, the research of various single component semiconductors, such as oxides^15,16^ and sulfide semiconductors^17,18^, has caught much attentions in the last decades. However, many reports have demonstrated that these single component semiconductors are photo-instability, fast recombination rate of electron hole pairs and wide band gaps^19^, exhibiting a significant limitation in achieving practical industrialization. To date, attempts have been made to overcome these disadvantages. For example, doping agents with non-metal ions, metalloids or ionic groups on the photocatalysts could effectively alter energy band structure and carrier separation^20^. It was found that N-doped CeO_x_ nanoparticles on g-C_3_N_4_ matrix presented a promoted photocatalytic activity due to formation of intimate interfacial contact^21^. B-doped BiOBr nanosheets could enhance the charge carrier separation efficiency, thereby enhancing the ability of accepting electrons from the valence band of BiOBr^22^. PO_4_ ionic group doped Bi_2_WO_6_ nanoplates demonstrated a significant regulation of their band structure, charge carrier separation efficiency and light absorbance for improving photocatalytic activity^20^. In addition, synthesis of heterojunction structure^23^ and decoration of noble metals^24^ in photocatalysts with enhanced visible light response and easy separation of electron-hole pairs have also induced extensive interests in recent years. For example, the formation of heterojunction structure on TiO_2_^25^, ZnO^26^ and some carbon matrix such as g-C_3_N_4_^27^, r-GO^28^ and carbon nanotubes^29^ presents an efficient separation of electrons and holes to facilitate photocatalytic activity. The decoration of noble metals, such as Au^30^, Ag^31^, Pt^32^ and Pd^33^ exhibits an improved light response to extend the light absorbance range.

Bi_2_MoO_6_, as one of the most important members in Aurivillius oxide family, is always at the cutting edge of research in photocatalytic degradation of organic pollutions^34^ and energy evolution^35^. The pure Bi_2_MoO_6_ with a relatively lower band gap (2.5–2.8 eV)^36^ presents an enhanced light response compared to some photocatalytic stars, such as TiO_2_ and ZnO with the band gaps of 3.2–3.3 eV^37^. However, due to their fast recombination efficiency of charge carriers, Bi_2_MoO_6_ usually presents a poor quantum yield that would greatly restrain their practical applications. So far, many attempts have been exploited to improve the photocatalytic performance of Bi_2_MoO_6_, such as doping lanthanide ions^38^ or noble metals^39^ to act as redox centers, the formation of heterojunction structures containing metal oxides^40^, metal sulfates^41^ or carbon materials^42^, etc.

Recently, g-C_3_N_4_ with its superior optical properties (band gap ≈ 2.7 eV) and photostability has been widely employed as a matrix to synthesize heterostructured photocatalysts^43^. The unique structure of covalently-linked sp^2^ bonded carbon network with the decoration of nitrogen atoms in g-C_3_N_4_ presents an excellent thermal and chemical stability. Moreover, the large scaffold structure could undoubtedly provide a large specific surface area for being anchored by other semiconductors and also could effectively prevent the particle aggregation. Nevertheless, the most important drawback of high recombination rate for the electron–hole pairs still largely inhibits their extensive applications. As such, the formation of heterojunctions by two semiconductors or anchoring noble metallic nanoparticles is rapid development in recent years, for example, the synthesis of g-C_3_N_4_ with semiconductors of TiO_2_^44^, ZnO^45^, NaNbO_3_^46^, Bi_2_WO_6_^47^ or TaON^48^ and the decoration of noble metals on g-C_3_N_4_ by Au^49^, Pt^50^, Ag^51^ or Pd^52^. However, the current studies have been focusing on synthesizing photocatalysts with two components. There are few attempts to investigate the noble metals being anchored on the heterojunctions, in which the optical structure is regulated by the synthesis of two semiconductors with a similar band gap and the electron-hole recombination efficiency is effectively suppressed by the noble metals doping.

In this work, Pt decorated g-C_3_N_4_/Bi_2_MoO_6_ heterostructured semiconductors are synthesized by one-step hydrothermal method to investigate their photocatalytic activity of MB degradation under visible light. Various amounts of Bi_2_MoO_6_ loading (10%, 20% and 50%) on g-C_3_N_4_ matrix are initially examined to demonstrate the optimal combination of the two semiconductors followed by Pt decoration. The structures of the synthetic composites are systematically characterized. The MB degradation and mineralization as well as the corresponding reaction kinetics (*k*_*obs*_) using the synthetic photocatalysts are comparatively investigated in detail. Quenching experiments by adding radical scavengers are also conducted to study the photocatalytic mechanisms.

## Results

### Structures and morphologies

**Figure 1:**
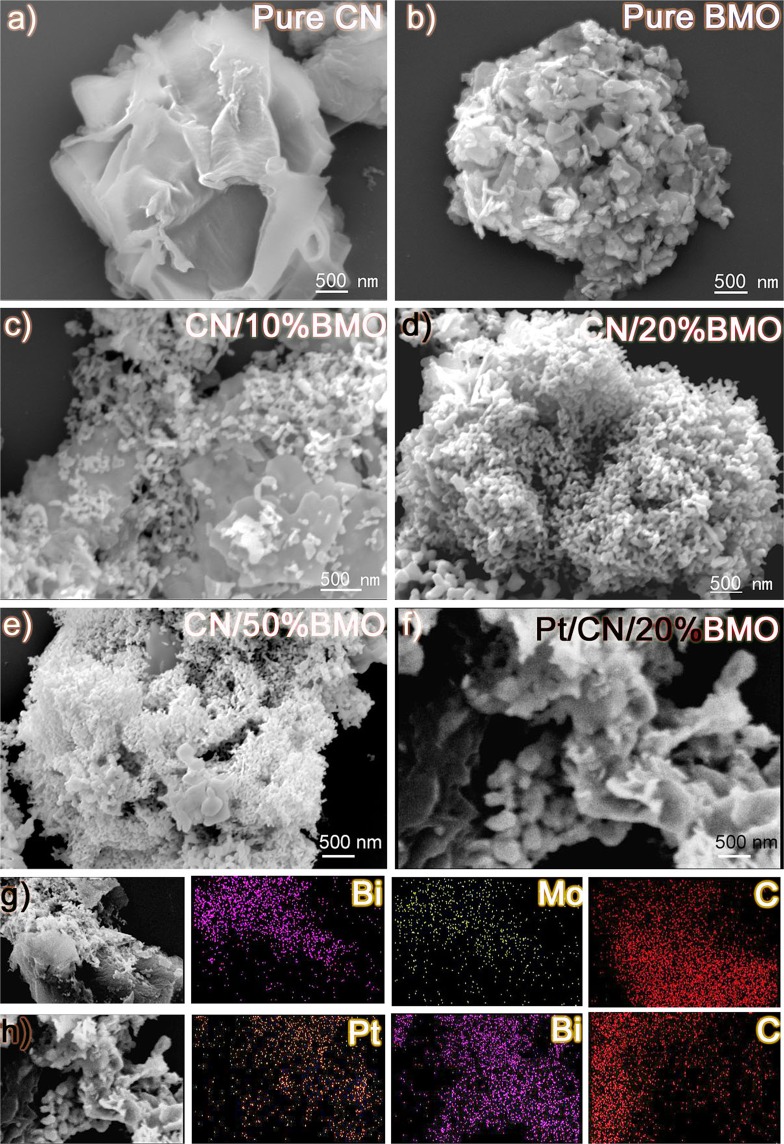
SEM images of (**a**) CN, (**b**) BMO, (**c**) CN/10%BMO, (**d**) CN/20%BMO, (**e**) CN/50%BMO, (**f**) Pt@CN/20%BMO and corresponding elemental mapping results of (**g**) CN/20%BMO and (**h**) Pt@CN/20%BMO.

Figure 1 shows scanning electron microscope (SEM) images of the as-prepared g-C_3_N_4_ (CN), Bi_2_MoO_6_ (BMO), g-C_3_N_4_/Bi_2_MoO_6_ composites with Bi_2_MoO_6_ contents of 10 wt.% (CN/10%BMO), of 20 wt.% (CN/20%BMO), of 50 wt.% (CN/50%BMO) and Pt decoration (Pt@CN/20%BMO), providing a direct view of surface morphologies of the photocatalysts. As shown in Fig. 1a, the obtained pure CN presents a flower-like structure with multiple wrinkled-layers, exhibiting a large surface area for further BMO and Pt nanoparticles decoration. Fig. 1b presents the pure BMO microplates with a laminar and irregular sheet-like microstructure. Notably, the BMO microplates have much smaller particle size than the CN, demonstrating great potential for them to anchor on the CN matrix. Fig. 1c–e show the morphologies of BMO and CN composites with a certain amount of BMO loading of 10%, 20% and 50%, respectively. Apparently, a denser BMO laminar sheet (Fig. 1d,e) is decorated on the wrinkled CN matrix compared to the CN/10%BMO loading in Fig. 1c. In addition, the size of the anchored BMO (Fig. 1c–e) is much smaller than that of the pure BMO (Fig. 1b) without altering the sheets-like morphology, providing more active sites for dye degradation. The smaller size of the anchored BMO is primarily ascribed to the initial adsorption of Bi^3+^ onto the CN according to the benefit of facile one-step hydrothermal synthetic method. The morphology of Pt@CN/20%BMO composites and atomic mapping images, e.g., Bi, Mo, C for CN/20%BMO and Pt, Bi, C for Pt@CN/20%BMO, are shown in Fig. 1f–h, providing a solid indication for the formation of Pt@CN/20%BMO heterojunctions.

**Figure 2:**
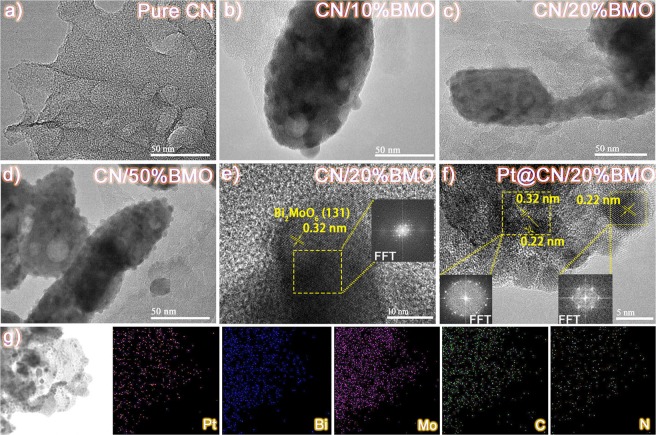
TEM images of (**a**) CN, (**b**) CN/10%BMO, (**c**) CN/20%BMO, (**d**) CN/50%BMO and HRTEM images of (**e**) CN/20%BMO, (**f**) Pt@CN/20%BMO (insets are FFT images of the selected areas) as well as the corresponding elemental mapping results of (**g**) Pt@CN/20%BMO.

Figure 2 shows transmission electron microscopy (TEM) and high resolution TEM (HRTEM) images of CN, CN/10%BMO, CN/20%BMO, CN/50%BMO and Pt@CN/20%BMO. The pure CN presents a wrinkled and layered morphology, as shown in Fig. 2a, which agrees with the SEM image (Fig. 1a) and other reports^53^. Fig. 2b–d imply the formation of CN and BMO heterojunctions and further reveal that the anchored BMO is in a sheet-like microplate. HRTEM images of CN/20%BMO and Pt@CN/20%BMO are presented in Fig. 2e,f. A lattice fringe with a space of 0.32 nm (Fig. 2e) corresponds to (131) plane of BMO, while the spacing of 0.22 nm (Fig. 2f) is ascribed to (111) plane of Pt with a particle size of ~5 nm. The fast fourier transform (FFT) images in the insets of Fig. 2e,f show that the single-crystalline structure of BMO is converted to poly-crystalline structure of Pt and BMO composites. Combining the elemental mapping images in Fig. 2g, this result further confirms the successful formation of Pt@CN/20%BMO heterojunctions.

**Figure 3:**
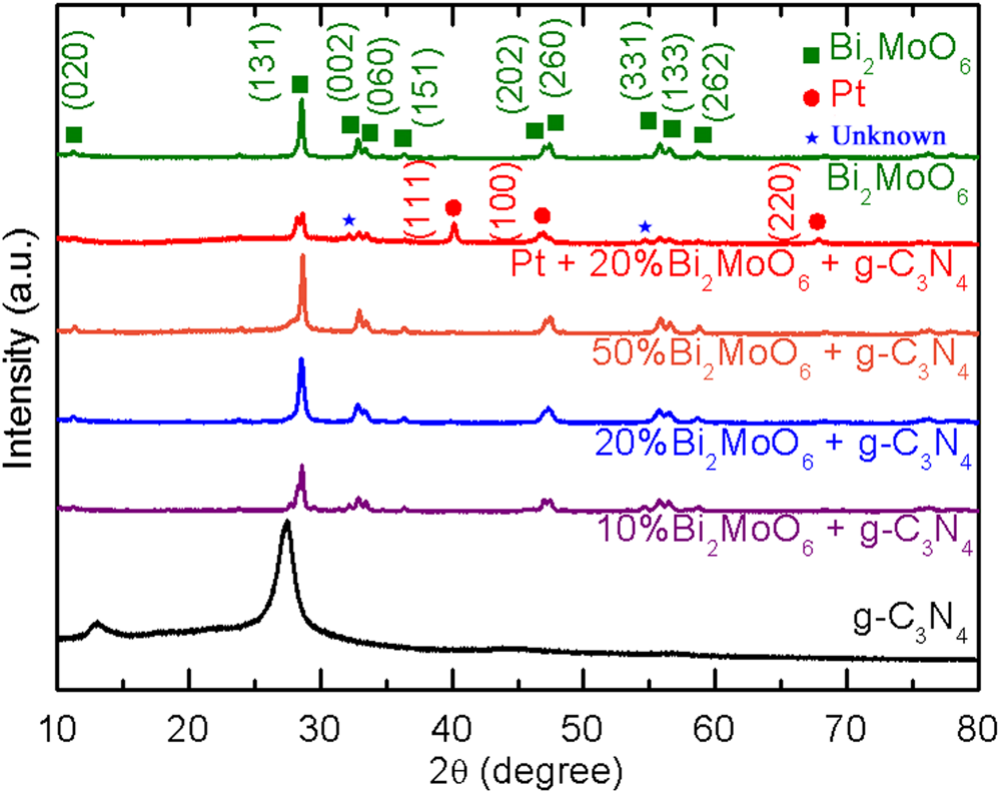
XRD characterization of the as-prepared photocatalysts.

The crystalline structures of the as-prepared samples are also investigated by X-ray diffraction (XRD) (Fig. 3). Two distinct peaks at 13.1° and 27.3° are observed on the CN curve, indicating (100) plane of tri-s-triazine group and (002) plane of aromatic laminar sheets, respectively^54^. XRD patterns are clearly characterized to confirm the orthorhombic crystalline structure of γ-BMO (JCPDS No. 21–0102)^40^. The strongest intensity at (131) plane is in accordance with the result of HRTEM image (Fig. 2c). For the CN/BMO composites, all the characterized diffraction peaks of BMO are remained, demonstrating the successful formation of heterojunctions. However, the peaks at (100) and (002) plane of CN are detected to be invisible and be overlapped with the peak at (131) plane of BMO, which is similar to other report^55^. For the Pt@CN/20%BMO composites, characterized diffraction peaks of Pt at 40.1, 46.9 and 67.9° indicate the (111), (100) and (220) planes while all the peaks of BMO are clearly observed, demonstrating Pt nanoparticles are decorated on the CN/BMO heterojunctions.

### Chemical states

**Figure 4:**
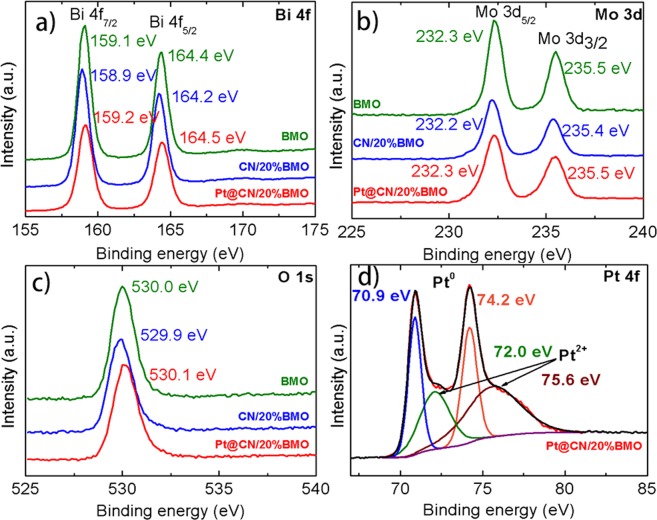
XPS results of the as-prepared photocatalysts: high-resolution spectra of (**a**) Bi 4 f, (**b**) Mo 3d, (**c**) O 1s and (**d**) Pt 4f.

**Figure 5:**
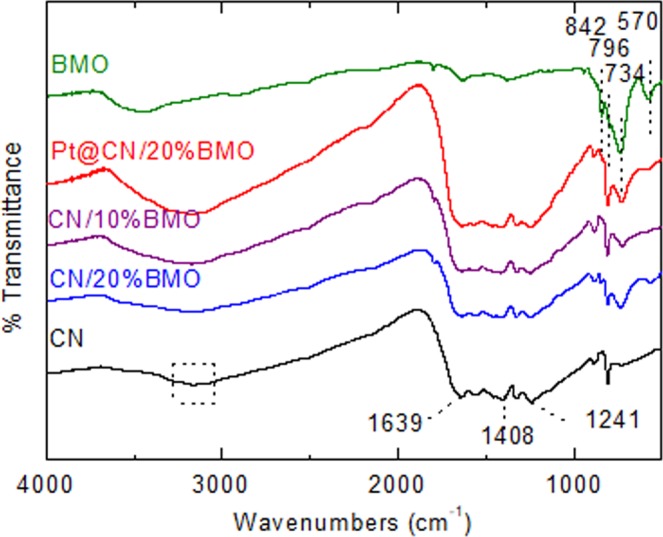
FTIR spectra of the as-prepared photocatalysts.

X-ray photoelectron spectroscopy (XPS) and fourier-transform infrared spectroscopy (FTIR) analyses have been conducted to confirm the surface chemical and valence states of the as-prepared samples. As shown in Figure S1, the elements including Pt, Bi, Mo, O, C and N with strong intensities are observed in the composites. No other impurity peaks are obtained. Fig. 4a shows the high-resolution spectra of Bi 4 f spectra for BMO, CN/20%BMO and Pt@CN/20%BMO. The bonding energies at 159.1 and 164.4 eV correspond to Bi 4f_7/2_ and Bi 4f_5/2_ of Bi^3+^, respectively^56^. For the Mo 3d spectra in Fig. 4b, the peaks located at 232.3 and 235.5 eV are attributed to Mo 3d_5/2_ and Mo 3d_3/2_ of Mo^6+^, respectively. In the O 1 s spectra (Fig. 4c), it is noted that the peak at 530.0 eV of pure BMO presents slight shifts to 529.9 eV and 530.1 eV for CN/20%BMO and Pt@CN/20%BMO samples, respectively. The shifted peaks in Bi 4 f and Mo 3d (Fig. 4a,b) indicate formation of the groups of Bi-O and Mo-O^57^. Such results further imply the formation of CN/BMO heterojunctions with Pt decoration. Fig. 4d shows the high resolution of Pt 4 f spectra. The characterized peaks at 70.9 and 74.2 eV of Pt^0^ are clearly observed on 4f_7/2_ and 4f_5/2_, respectively. The peaks at 72.0 and 75.6 eV are attributed to Pt^2+^, indicating the formation of Pt-O^58^ and further demonstrating the partial pre-adsorption of Pt^2+^ onto the CN matrix during the facile one-step hydrothermal synthetic method. Fig. 5 shows the FTIR results of the as-prepared composites. For the pure CN, a group of characterized peaks are clearly observed in the range from 1241 to 1639 cm^−1^. In detail, the peaks located at 1241,1325 and 1408 cm^−1^ are attributed to the stretching vibration mode of C-N bonds with the aromatic structure, while the peak at 1639 cm^−1^ can be ascribed to the stretching vibration mode of C≡N^59^. For the pure BMO, the characterized peaks located at 570 and 734 cm^−1^ indicate the bending vibration of the octahedral structure and the asymmetric stretching mode in MoO_6_, respectively, while the wavenumbers at 796 and 842 cm^−1^ correspond to the Mo-O symmetric and asymmetric stretching vibration modes in the MoO_6_ octahedral structure^59^. Clearly, all the characterized peaks in pure CN and BMO are presented in the CN/20%BMO and Pt@CN/20%BMO composites, demonstrating the successful formation of the photocatalysts. Such a result is in great agreement with the aforementioned SEM, TEM and XRD analyses.

### Optical property

**Figure 6:**
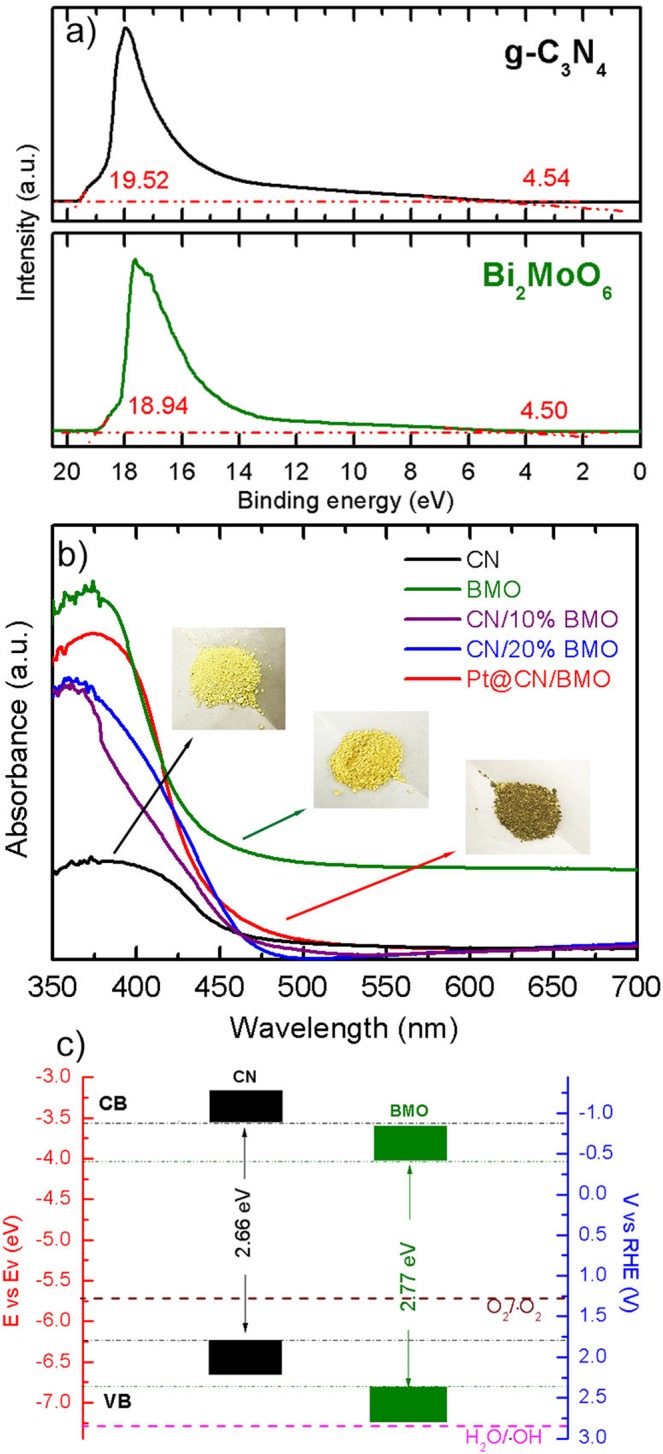
(**a**) UPS spectra, (**b**) UV-Vis DRS spectra, and (**c**) the estimated band gap structures of the CN and BMO.

The optical property plays a significant role in revealing the band gap structures of heterojunction photocatalysts. Fig. 6a shows the ultraviolet photoelectron spectroscopy (UPS) spectra of the as-prepared CN and BMO composites to indicate the Fermi (*E*_*Fermi*_) and cut-off (*E*_*cut-off*_) energy states by estimating the intersection values. Notably, the valence band energy (*E*_*VB*_), which is equivalent to the ionization potential (*φ*), is calculated by the equation below (Equation 1)^54^:

1 $$\phi =hv-{E}_{cut \mbox{-} off}+{E}_{Fermi}$$

whereas *hv* is the photon energy from He I source as 21.22 eV.

The values of *φ* are then calculated as 6.24 and 6.78 eV for the as-prepared CN and BMO, respectively, suggesting the electrons would transfer from CN to BMO (*φ*_*CN*_ < *φ*_*BMO*_) after the formation of heterojunction structure^40^. In order to find out the position of conduction band (*E*_*CB*_), the band gap energy (*E*_*g*_) is estimated by UV-Vis diffuse reflectance spectrum (UV-DRS) spectra as shown in Fig. 6b using the equation below (Equation 2)^60^,

2 $${E}_{g}(eV)=1240/{\lambda }_{g}$$

whereas the absorption edge (*λ*_*g*_) is observed by the intersection values of the tangent and the wavelength axis.

The values of *E*_*g*_ and *E*_*CB*_ of the as-prepared CN and BMO samples can be subsequently calculated as 2.66, 2.77 eV (*E*_*g*_) and 3.58, 4.01 eV (*E*_*CB*_ = *E*_*VB*_ − *E*_*g*_), respectively. In the expression of reversible hydrogen electrode (RHE) vs. volts (V) (0 V in RHE is equivalent to −4.44 eV in energy potential^61^), the *E*_*CB*_ can be positioned at −0.86 and −0.43 V of the CN and BMO composites, respectively. Fig. 6c presents the estimated band structures of the prepared CN and BMO samples according to the above calculation. Due to the similar band structures of CN and BMO, the electrons in the CB and the holes in the VB would be promoted from the CN to BMO and BMO to CN, respectively. The electronic motion in the modified band structure of CN/BMO heterostructures would be improved, and more importantly, the inclusion of Pt nanoparticles as electron mediators anchoring on the CN/BMO heterostructures would further promote the electron transfer. In addition, the observation of the improved absorption intensity in the visible light region for the Pt@CN/20%BMO photocatalyst indicates that the band structure could be effectively altered to enhance the visible light response and charge transformation. The improved absorption intensity and slight red shift of Pt@CN/20%BMO photocatalyst can be ascribed to the localized surface plasmon resonances (LSPRs) of Pt nanoparticles^24^, which would enhance the photocatalytic activity for dye degradation.

The heterogeneous photocatalytic activity is also significantly influenced by the specific surface area of photocatalysts due to the provision of surface-reactive sites. Figure S2 presents the nitrogen adsorption/desorption isotherms of the as-prepared samples. Clearly, strong N_2_ adsorption/desorption curves are observed in the pressure range of 0.1–0.9, indicating the capillary condensation in large mesopores exists in all the samples^55^. As shown in Figure S2 inset, the Brunauer-Emmett-Teller (BET) specific surface area of Pt@CN/20%BMO with 46.09 m^2^/g presents significant improvement compared to the pure BMO with 10.79 m^2^/g and CN/20%BMO with 39.92 m^2^/g, respectively. In addition, the Pt@CN/20%BMO with a total pore volume of 1.092 cm^−3^/g (Barrett-Joyner-Halenda (BJH) process) would provide much more active sites compared to the other samples. Such results are in accordance with the above SEM analysis. The Pt nanoparticles and BMO laminar sheets are densely distributed on the wrinkled CN matrix that would lead to a higher photocatalytic activity for the following dye degradation. Figure S3 shows the thermogravimetric analysis (TGA) results of the as-prepared samples. It is noted that the TGA curve of the pure CN presents a sharp weight loss in the temperature range from 500 to 680 °C and nearly no CN remaining after 680 °C, while there is only 1.1% weight loss for the pure BMO samples due to the slight adsorption and activation of atmospheric oxygen^55^. However, the significant weight loss of CN/20%BMO and Pt@CN/20%BMO is shifted to the temperature range from 400 to 580 °C and 380 to 570 °C, respectively. Surprisingly, the obtained ratio between BMO and CN from TGA result is higher than the theoretical value of 20%, which is mainly attributed to the existence of BMO and Pt would promote the oxidation of CN and the adsorption of atmospheric oxygen. Similar performance has been reported in g-C_3_N_4_/Bi_2_MoO_6_^55^ and g-C_3_N_4_/SmVO_4_^62^ heterojunction photocatalysts.

### Photocatalytic activity

**Figure 7:**
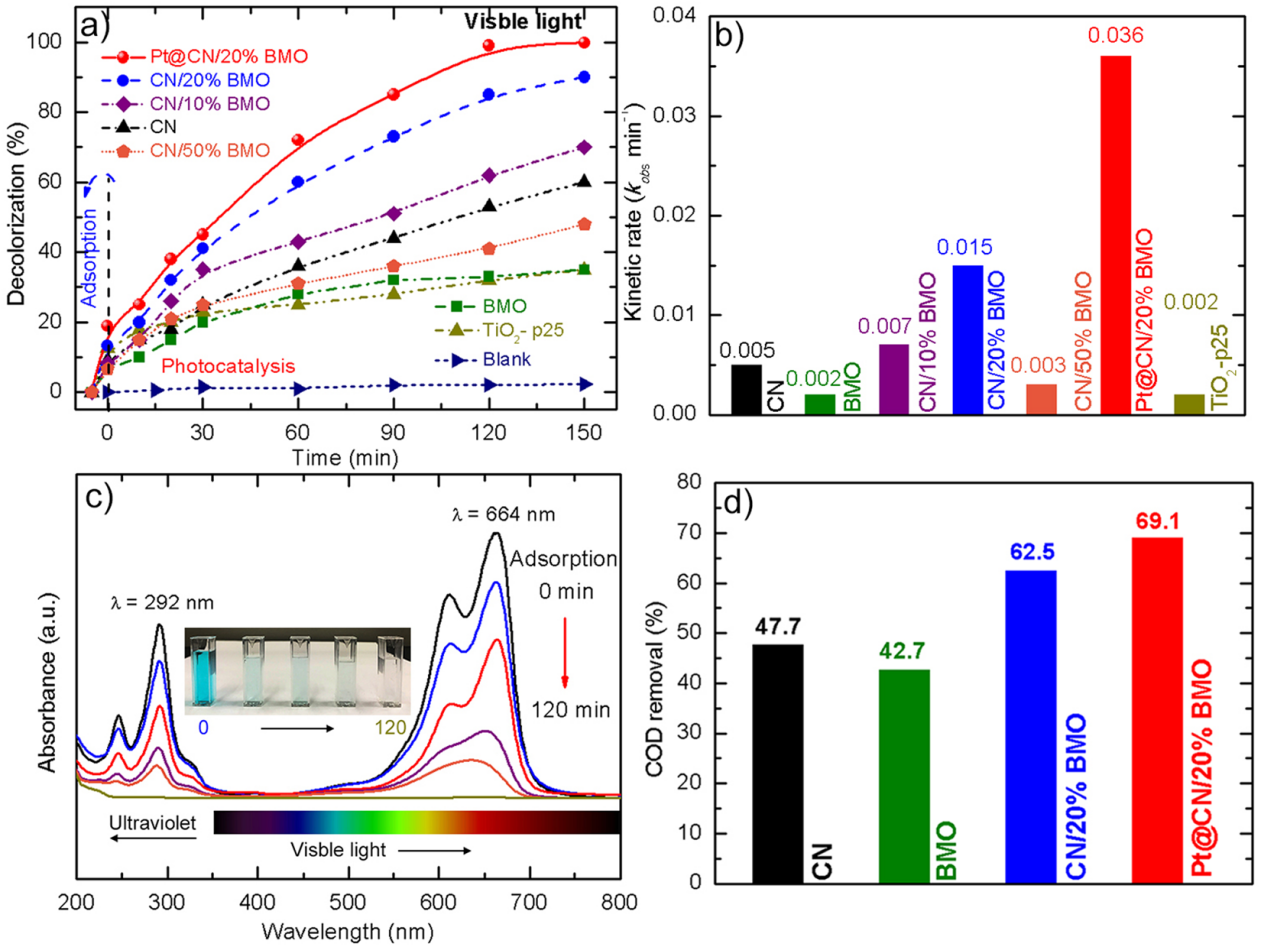
(**a**) photocatalytic MB degradation and (**b**) corresponding kinetic rates (*k*_*obs*_) of the as-prepared samples, (**c**) UV-Vis spectra of color removals for Pt@CN/20%BMO photocatalysts and (**d**) COD removals at 150 min of as-prepared samples under visible light.

The photocatalytic activity of the as-prepared samples was examined by the degradation of MB dye under visible light (λ≥420 nm). As shown in Fig. 7a, the MB dye without the addition of catalysts presents negligible change under visible light irradiation even for 150 min. However, when adding the synthetic CN/BMO composites, the MB degradation efficiency has a significant increase compared to the pure BMO or CN, demonstrating the superior photocatalytic activity of the as-prepared heterojunction structures. In order to find out the optimal weight percentage of BMO, the CN/BMO composites with BMO loading of 10%, 20% and 50% are investigated for the MB degradation. It is interesting that the photocatalytic activity is remarkably improved when increasing the BMO contents from 0% to 20%, while 90% of MB degradation for CN/20%BMO compared with only 38% of MB degradation for the pure BMO at 150 min. However, when 50% of BMO is loaded, the MB degradation presents a significant decrease to be even lower than the pure CN. It is owing to the excessive BMO microplates could cover the surface of CN so that to reduce the photon adsorption in the synthetic heterostructural CN/BMO photocatalysts, and moreover, the formation of heterojunction could be suppressed by the inclusion of excessive BMO microplates due to self-agglomerate phenomenon^63^, which is in good accordance with the SEM image in Fig. 1e. Such performance suggests that the CN/BMO composite with 20% BMO loading exhibits the optimal photocatalytic activity for dye degradation. On this basis, further study is investigated for the Pt@CN/20%BMO composites. Clearly, the Pt decorated photocatalysts present a significant enhancement for the MB degradation compared to all the others, whereas nearly 100% dye degradation is achieved at 120 min, indicating that its heterojunction structure is successfully regulated by the facile hydrothermal synthesis. The corresponding kinetic rates (*k*_*obs*_) of the as-prepared samples for MB degradation are calculated by the pseudo-first-order kinetics model (Equation 3) as shown in Fig. 7b.

3 $$\mathrm{ln}({C}_{0}/C)={k}_{obs}t$$

whereas *k*_*obs*_ is the kinetic rate constant; *C*_0_ is the original concentration of dye; *C* is the dye concentration at time *t*.

Notably, the Pt@CN/20%BMO composites with the *k*_*obs*_ = 0.036 min^−1^ presents 7 times and 18 times higher photocatalytic activity than the pure CN with *k*_*obs*_ = 0.005 min^−1^ and BMO with *k*_*obs*_ = 0.002 min^−1^, respectively. In addition, the photoactivity of the synthetic Pt@CN/20%BMO composites is much promoted than the commercial p-25 with *k*_*obs*_ = 0.002 min^−1^ and also other state-of-the-art ternary photocatalysts under visible light (Table 1), demonstrating their excellent catalytic property in wastewater remediation. Fig. 7c presents the UV-Vis spectra of MB degradation using 0.5 g/L of Pt@CN/20%BMO photocatalyst at different time intervals. It can be observed that the characterized peaks at *λ* = 292 nm (triazine group) and *λ* = 664 nm (heteropoly aromatic linkage) of MB gradually decrease to be invisible from 0 min to 120 min, indicating the organic components in the dye are decomposed to nontoxic NO_3_^−^, SO_4_^2−^, H_2_O and CO_2_^3,64^. The slight blue shift (towards blue end in the spectrum) of the absorbance in MB dye from 664 nm to 634 nm is likely ascribed to the initial degradation of auxochrome (-CH_3_), resulting in a slight color change during photocatalytic activity^65^. The inset of Fig. 7c presents a solid evidence of the visible color change during MB degradation.

**Table 1:** Comparison of ternary photocatalysts for degradation of organic pollutants under visible light.

SC 1	SC 2	Metallic mediators	Light source (Power, W)	Organic pollutants	Degradation (%) and Time (h)	Ref.
CdS	TiO_2_	Au	LP Hg lamp (20)	MB	72 and 2	^70^
g-C_3_N_4_	BiPO_4_	Au	Xe lamp (300)	MO	88 and 2.66	^71^
MoS_2_	Ag_3_PO_4_	Ag	Solar Xe arc lamp (35)	PhOH	95 and 2	^72^
g-C_3_N_4_	WO_3_	Cu, Ag, Au	Xe arc lamp (500)	4-NPhOH	100 and 2	^73^
In_2_S_3_	Ag_2_CrO_4_	Ag	Xe arc lamp (300)	MO	65.3 and 2	^74^
ZnS	Ag_3_PO_4_	Ag	Xe lamp (350)	MB	82 and 2	^75^
g-C_3_N_4_	Ag_2_CrO_4_	Ag	HP Xe Lamp (500 W)	2,4-DCP	94 and 2	^76^
CdS	BiOCl	Au	Xe lamp (300)	SD	100 and 4	^77^
g-C_3_N_4_	Bi_2_MoO_6_	CNT	Xe lamp (500)	2,4-DBP	68.8 and 2	^78^
g-C_3_N_4_	Bi_2_MoO_6_	Pt	Xe lamp (300)	MB	100 and 2	This work

Fig. 7d shows the chemical oxygen demand (COD) removals of the as-prepared samples at 150 min under visible light irradiation. Notably, the pure CN and BMO with the COD removals of 47.7% and 42.7% present a relatively poor performance compared with the Pt@CN/20%BMO composites with the COD removal of 69.1%, further implying the synthetic Pt@CN/20%BMO photocatalyst can effectively mineralize the MB dye into H_2_O and CO_2_ during the photocatalytic performance. Such a result is in good accordance with the above analyses of the promoted regulation of electron transfer, specific surface area and surface morphology, etc.

It is well accepted that the progress of dye molecule decomposition is mainly due to the generation of the active species during photocatalysis. To identify these dominant active species, various radical scavengers including IPA, CrO_3_, SO and BQ are employed to prove the generation of ^•^OH, e^−^, h^+^ and ^•^O^2−^, respectively^35,59,66^. As shown in Figure. S4, the addition of IPA causes a decrease in MB degradation compared to the only addition of Pt@CN/20%BMO photocatalysts, suggesting the ^•^OH radicals are gradually formed during this photocatalytic activity. In comparison, the MB degradation efficiency is more affected by the addition of CrO_3_, SO and BQ, leading to the efficiency sharply decreases to nearly 50% at 150 min. Such performance indicates that the generated e^−^, h^+^ and ^•^O^2−^ are the dominant oxidants for MB degradation in this work.

## Discussion

**Figure 8:**
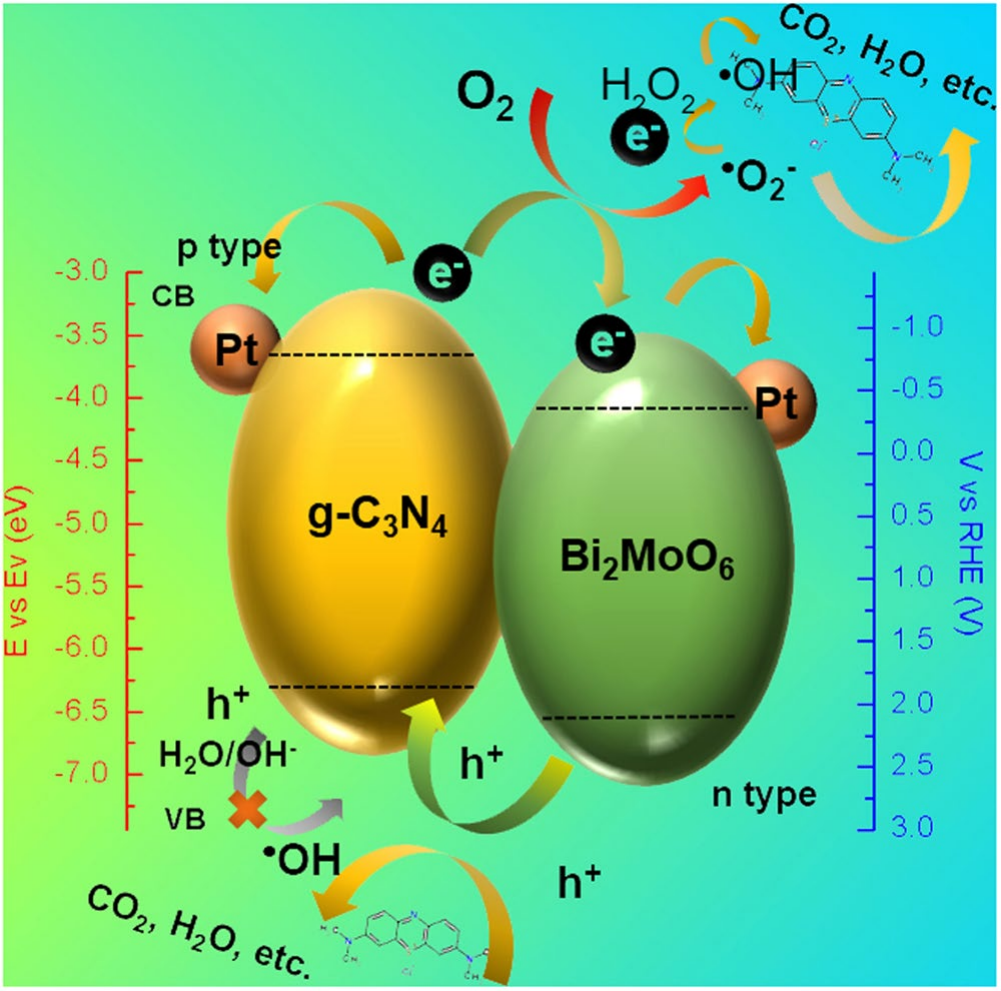
Schematic graph of the proposed photocatalytic mechanism for the synthetic Pt decorated CN/BMO heterojunctions.

Fig. 8 shows the proposed photocatalytic mechanism of the synthetic heterojunctions. Considering the above band gap analysis in Fig. 6, the photo-induced h^+^ with an energy of 2.34 V at VB presents lower energy state than the ^•^OH of 2.70 V, leading to the ^•^OH cannot be directly activated from H_2_O during the photocatalysis. The existing ^•^OH is produced by the reaction of e^−^ and the gradually generated H_2_O_2_^59^. This is the reason why sole ^•^OH plays partially synergistic dye degradation in this work. Therefore, the h^+^ with strong oxidation ability is directly employed for decomposing the MB molecule. In comparison, the potential of e^−^ with the value of -0.86 V at CB is much negative than the ^•^O^2−^ with the value of 1.23 V, resulting in easy reduction of dissolved O_2_ to ^•^O^2−^ thereby degrading the organic matters. More importantly, the decorated Pt nanoparticles in this work play a significant role to serve as a solid-state electron mediator. The generated e^−^ on the CB of g-C_3_N_4_ in the heterojunctions is easily transferred to the Pt particles and Bi_2_MoO_6_ microplates that would effectively promote electron transfer and suppress the electron-hole recombination efficiency^55^. In addition, the produced h^+^ also could migrate from Bi_2_MoO_6_ to g-C_3_N_4_ in the heterojunctions, which also provides significant reduction of recombination efficiency. Therefore, the photocatalytic dye degradation efficiency of Pt@CN/20%BMO is more enhanced than those of the pure BMO, CN and even for the CN/BMO composites.

In this work, Pt nanoparticles were successfully decorated the Bi_2_MoO_6_ (BMO) and g-C_3_N_4_ (CN) heterojunctions by a one-step facile hydrothermal method. It is confirmed that the Pt decorated CN and BMO heterojunctions with 20% weight percent of BMO (Pt@CN/20%BMO) with a large specific surface area and the modified band gap present a much higher photocatalytic activity for MB degradation compared to the pure CN and BMO as well as the CN/BMO composites. The decorated Pt nanoparticles on the composites could act as a solid-state electron mediator that plays a significant role to promote electron transfer and suppress the recombination rate of electron-hole pairs, thus improving the photostability. In addition, the observation of the improved absorption intensity in the visible light region for the Pt@CN/20%BMO photocatalyst indicates that the band structure could be effectively altered to enhance the visible light response and charge transformation. As a consequence, the photocatalytic activity could be largely improved by the use of the simply synthetic Pt@CN/20%BMO composites, providing new insights into the future work on the synthesis of green photocatalysts.

## Methods

### Materials

Bismuth nitrate pentahydrate (Bi(NO_3_)_3_·5H_2_O), ammonium molybdate tetrahydrate ((NH_4_)_6_Mo_7_O_24_·4H_2_O), potassium tetrachloroplatinate (K_2_PtCl_4_) and melamine were purchased from Strem Chemicals, INC (USA). Chromium trioxide (CrO_3_), sodium oxalate (SO, Na_2_C_2_O_4_), 1,4-benzoquinone (BQ) and isopropanol (IPA) supplied from J&K Scientific Ltd., (China) were used throughout this work as electron (e^−^), hole (h^+^), superoxide radicals (^•^O_2_^−^), and hydroxyl radicles (^•^OH) scavengers, respectively. The methylene blue (MB) dye was supplied from Sigma Aldrich. Other chemicals such as HNO_3_ (0.5 M), NaOH (0.5 M), distilled water, were all of the analytical grades.

### Synthesis of g-C_3_N_4_/Bi_2_MoO_6_/Pt heterojunctions

Melamine was directly heated to prepare the g-C_3_N_4_ (CN) powders in a semi-closed system by a reported method^55^. Typically, 10 g of melamine in a covered crucible under ambient pressure in air was heated to 520 °C in a muffle furnace at a heating rate of 5 °C/min and then retained for 140 min.

The Bi_2_MoO_6_ (BMO) microplates were synthesized by a simply hydrothermal process. In detail, 1.00 mmol (0.485 g) of Bi(NO_3_)_3_·5H_2_O and 0.07 mmol (0.088 g) of (NH_4_)_6_Mo_7_O_24_·4H_2_O were dissolved in 40 mL distilled water followed by vigorous stirring for 60 min. After being another sonicated 10 min, the resultant solution was transferred into a 50 mL Teflon-lined stainless-steel autoclave and heated up to 160 °C for overnight with naturally cooling to room temperature. Afterwards, the solid samples were obtained by filtration, which were washed with distilled water by 3 times. The collected powders were further dried at 80 °C in air overnight then annealed in a muffle furnace at 400 °C for 3 h at a heating rate of 5 °C/min.

g-C_3_N_4_/Bi_2_MoO_6_ and Pt decorated g-C_3_N_4_/Bi_2_MoO_6_ heterojunctions were synthesized by one step facile hydrothermal method. For the g-C_3_N_4_/Bi_2_MoO_6_ heterojunctions, 0.485 g of Bi(NO_3_)_3_·5H_2_O and 0.088 g of (NH_4_)_6_Mo_7_O_24_·4H_2_O were firstly dissolved in 40 mL distilled water under stirring for 60 min. Afterwards, different weights of g-C_3_N_4_ (i.e. 2.7 g, 1.2 g and 0.3 g) were gradually added into the mixed solvent and stirred for another 30 min. The weight percentages of Bi_2_MoO_6_ in g-C_3_N_4_/Bi_2_MoO_6_ composites were 10%, 20% and 50%, denoted as CN/10%BMO, CN/20%BMO and CN/50%BMO in this work. For the preparation of Pt decorated g-C_3_N_4_/Bi_2_MoO_6_ heterojunctions, 0.157 mmol (0.065 g) of K_2_PtCl_4_ was gradually dropped into the pre-prepared mixed solution containing Bi(NO_3_)_3_·5H_2_O and (NH_4_)_6_Mo_7_O_24_·4H_2_O for stirring another 30 min. Then 1.2 g g-C_3_N_4_ (20%Bi_2_MoO_6_) was added into the solution for vigorous stirring in 30 min. The Pt decorated CN/20%BMO composites was denoted as Pt@CN/20%BMO. After being sonicated for 10 min, the mixed composites were heated, filtrated, dried and annealed as the same procedures of the abovementioned Bi_2_MoO_6_ preparation (the annealed temperature of Pt decorated heterojunction was at 350 °C with the same heating rate of 5 °C/min).

### Characterizations

The SEM (JEOL JSM-820) and TEM (JEOL TEM 2100F FEG) as well as HRTEM equipped with energy-dispersive X-ray spectroscopy (EDS) were employed for characterizing the surface morphology and inner structure of the as-prepared samples. The crystalline phases of the samples were examined by XRD (Rigaku SmartLab) using Cu Kα radiation. XPS measurements were performed on a VG ESCALAB 220i-XL surface analysis system to record the surface atomic distribution of the as-prepared powders. The structural characterization of the samples was collected using FTIR (Thermo Scientific iS50). The UPS (VG ESCALAB 220i-XL) and UV-Vis DRS (PerkinElmer Lambda 750) using BaSO_4_ as the reference were employed to characterize the electronic levels and band structures of the as-prepared samples. The specific surface area was recorded by BET on a Micromeritics, ASAP2020 gas sorption analyzer at 77 K. TGA was carried out on a TGA Q50 instrument by heating from 30 to 800 °C at a heating rate of 10 °C/min under nitrogen protection. The UV-Vis spectrometer (PerkinElmer Lambda2S) was employed for measuring the dye absorbance.

### Photocatalytic activity

In the photocatalytic experiments, a specific mass of the as-prepared photocatalysts was dispersed in 100 ml of MB solution with 10 ppm concentration (10 mg/L). The MB solution with catalysts was initially stirred for 60 min in darkness to achieve equilibrium adsorption, followed by a direct irradiation under a source of 300 W Xeon simulated solar light lamp with an ultraviolet cut off filter (≥420 nm) to provide visible light. Afterwards, 4 ml of the MB solution were taken at different predetermined time intervals and separated in a MIKRO 185 centrifuge at 10000 rpm for 2 min to remove the remaining solid catalysts. Then the samples were recorded by the UV-Vis spectrometer and COD determination with reference to dichromate method (HJ 828–2017 standard). The wavelength of light absorbance *λ*_*max*_ of MB solution was at 664 nm^67,68^. The dye degradation efficiency were evaluated by the following Equation 3 ^69^:

4 $$X=({C}_{0}-C)/{C}_{0}\times 100 \% $$

whereas *C*_0_ and *C* are the initial concentration and the concentration at time *t* of MB dye, respectively.

## Supplementary information

SREP-18-40260_R1_Supporting Information_R1
