# Supplementary material for: Pt nanoparticles decorated heterostructured g-C3N4/Bi2MoO6 microplates with highly enhanced photocatalytic activities under visible light
Source: Sci Rep. 2019 May 21;9:7636. doi: 10.1038/s41598-019-42973-6 (PMC6529451; doi:10.1038/s41598-019-42973-6)
Supplement: Supplementary file 1 — SREP-18-40260_R1_Supporting Information_R1 [file 41598_2019_42973_MOESM1_ESM.docx]

**Supporting Information**

**Pt nanoparticles decorated heterostructured g-C_3_N_4_/Bi_2_MoO_6_ microplates with highly enhanced photocatalytic activities under visible light**

*Z. Jia1,* *F. Lyu^1^, L.C. Zhang^2^, S. Zeng^3,4^, S.X. Liang^2^, Y.Y. Li^3,4,5^, J. Lu^1,5^^[[1]](#footnote-1)^**

***^1^Hong Kong Branch of National Precious Metals Material Engineering Research****,* *Department of Mechanical and Biomedical Engineering,* *City University of Hong Kong,* *83 Tat Chee Avenue, Kowloon, Hong Kong, China*

*^2^School of Engineering, Edith Cowan University, 270 Joondalup Drive, Joondalup, Perth, WA 6027, Australia*

*^3^Center of Super-Diamond and Advanced Films (COSDAF), City University of Hong Kong, Kowloon, Hong Kong, China*

*^4^Department of Physics and Materials Science, City University of Hong Kong, Kowloon, Hong Kong, China*

*^5^Centre for Advanced Structural Materials, City University of Hong Kong, Shenzhen Research Institute, 8 Yuexing 1st Road, Shenzhen Hi-Tech Industrial Park, Nanshan District, Shenzhen, China*

The authors declare no competing financial interest.

*Email: jianlu@cityu.edu.hk (J. Lu)


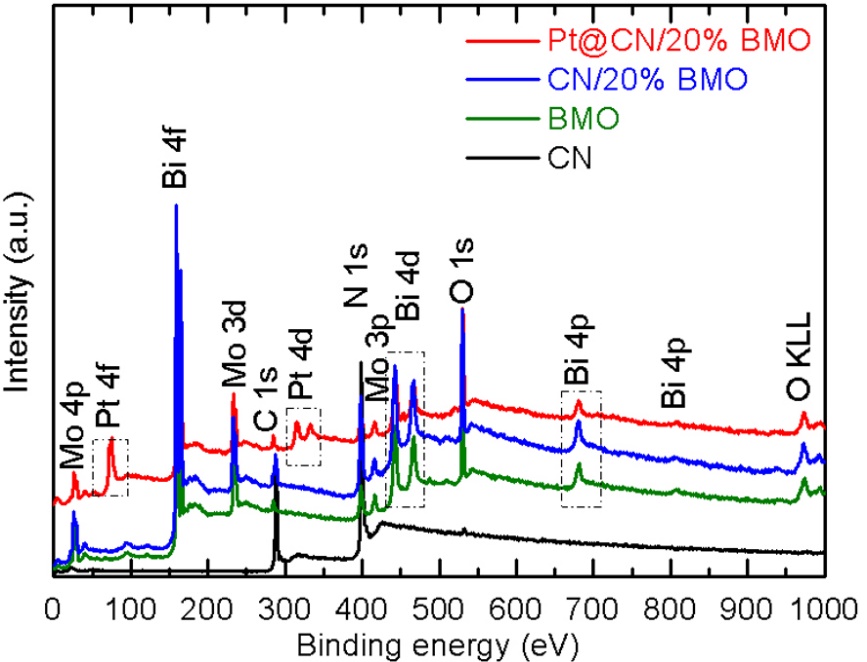


**Figure S1.** XPS results of the as-prepared photocatalysts in full range


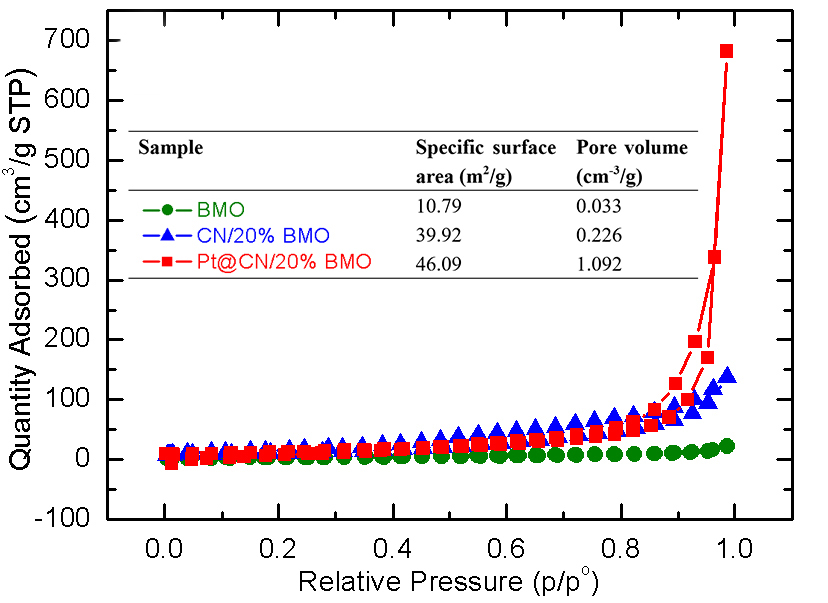


**Figure S2.** Nitrogen adsorption/desorption of the as-prepared samples(inset is the BET specific surface area (m^2^/g) and pore volume (cm^-3^/g) by BJH method)


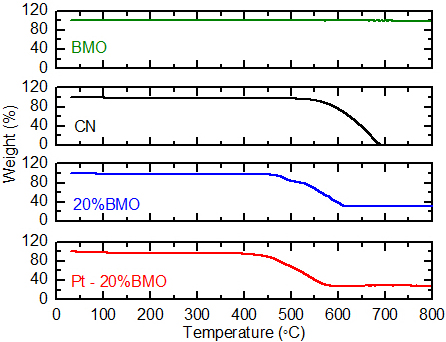


**Figure S3.** TGA analysis of the as-prepared samples


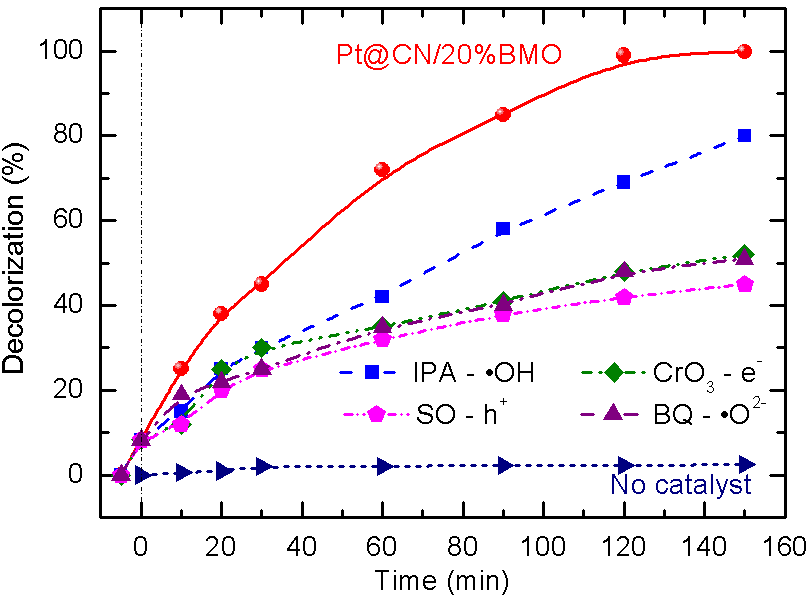


**Figure S4.** Photocatalytic degradation of MB on Pt@CN/20%BMO photocatalysts with and without adding quenching agents of IPA, SO, CrO_3_ and BQ under UV-Vis light

1. *Corresponding author: Prof. Jian Lu. Tel: +852 **3442 6847; email address:** jianlu@cityu.edu.hk [↑](#footnote-ref-1)
